# Relieving Cost of Epidemic by Parrondo's Paradox: A COVID‐19 Case Study

**DOI:** 10.1002/advs.202002324

**Published:** 2020-11-05

**Authors:** Kang Hao Cheong, Tao Wen, Joel Weijia Lai

**Affiliations:** ^1^ Science, Mathematics and Technology Cluster Singapore University of Technology and Design 8 Somapah Rd, S487372 Singapore

**Keywords:** COVID‐19, epidemic strategies, game theory, modeling, pandemics, Parrondo's paradox, population dynamics

## Abstract

COVID‐19, also known as SARS‐CoV‐2, is a coronavirus that is highly pathogenic and virulent. It spreads very quickly through close contact, and so in response to growing numbers of cases, many countries have imposed lockdown measures to slow its spread around the globe. The purpose of a lockdown is to reduce reproduction, that is, the number of people each confirmed case infects. Lockdown measures have worked to varying extents but they come with a massive price. Nearly every individual, community, business, and economy has been affected. In this paper, switching strategies that take into account the total “cost” borne by a community in response to COVID‐19 are proposed. The proposed cost function takes into account the health and well‐being of the population, as well as the economic impact due to the lockdown. The model allows for a comparative study to investigate the effectiveness of various COVID‐19 suppression strategies. It reveals that both the strategy to implement a lockdown and the strategy to maintain an open community are individually losing in terms of the total “cost” per day. However, switching between these two strategies in a certain manner can paradoxically lead to a winning outcome—a phenomenon attributed to Parrondo's paradox.

## Graphical abstract

This work reveals that both the lockdown strategy and the open community strategy are individually losing in terms of the total “cost” per day. However, switching between these two strategies in a certain manner can paradoxically lead to a winning outcome—a phenomenon attributed to Parrondo's paradox. This is one of the first studies linking Parrondo's paradox to epidemiology.

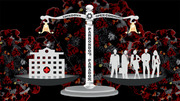

## 1 Introduction

The outbreak of coronavirus disease 2019 (COVID‐19) has spread rapidly across the globe at an alarming pace, causing considerable anxiety and fear among the general public.^[^ ^1^, ^2^, ^3^ ^]^ The epidemic has witnessed an exponential growth in infections without any proper strategy to control the spread.^[^ ^4^ ^]^ The issues involved in the epidemic are both nuanced and complex. Approaching these issues from multiple fields of expertise such as machine learning,^[^ ^5^, ^6^, ^7^, ^8^ ^]^ complexity science,^[^ ^9^, ^10^, ^11^ ^]^ network science,^[^ ^12^, ^13^, ^14^ ^]^ and uncertainty measure^[^ ^15^, ^16^, ^17^ ^]^ will help accelerate us toward solutions. Early lessons from some countries have shown that, by not imposing a lockdown, increases in the numbers of infected individuals and deaths^[^ ^18^, ^19^, ^20^ ^]^ are inevitable. Other than the rampant spread of COVID‐19, populations are also coping with other substantial health and psychological stresses.^[^ ^21^, ^22^ ^]^ This will put a strain on healthcare services, especially for countries with less developed facilities ill‐equipped to manage the sudden spikes in hospital admission. Irrefutably, an absolute lockdown reduces the opportunities for individuals to come into close contact with one another, thereby limiting the disease transmission.^[^ ^23^ ^]^ While an absolute lockdown is effective in curbing an epidemic,^[^ ^9^, ^24^, ^25^ ^]^ it will paralyze economic activity across the world as factories shut, bringing manufacturing to a halt, and individual activities are hampered.^[^ ^26^, ^27^ ^]^ This will have a huge impact on the economy and individual purchasing power.^[^ ^28^, ^29^ ^]^ That is to say, lockdown strategy has benefits in alleviating the spread of the virus, but it will decimate the economy. When considering the health and well‐being of the population, as well as economic impacts, the two strategies—a) keeping communities open and b) implementing lockdown—are both losing strategy when considered individually. We then ask an important question: can both losing strategies be combined in a certain manner, leading to a winning outcome, defined in our model as an outcome having a falling daily “cost” over time? In this paper, we seek to answer this question by modeling the population using Parrondo's paradox with the view of relieving cost of the epidemic by a switching strategy. We will explore how the combination of two losing strategies can lead to a winning outcome, similar to the framework of Parrondo's paradox.^[^ ^30^ ^]^ Parrondo's paradox was first conceptualized as an abstraction of flashing Brownian ratchets,^[^ ^31^, ^32^, ^33^ ^]^ wherein diffusive particles exhibit unexpected drift when exposed to alternating periodic potentials. It has since been applied across a wide range of disciplines in the physical sciences and engineering‐related fields,^[^ ^34^, ^35^ ^]^ such as diffusive and granular flow dynamics,^[^ ^36^, ^37^ ^]^ information thermodynamics,^[^ ^38^, ^39^, ^40^ ^]^ chaos theory,^[^ ^41^, ^42^, ^43^, ^44^, ^45^, ^46^, ^47^ ^]^ switching problems,^[^ ^48^, ^49^, ^50^ ^]^ and quantum phenomena.^[^ ^51^, ^52^, ^53^, ^54^, ^55^, ^56^, ^57^ ^]^ The paradox has also found numerous applications in life science,^[^ ^58^, ^59^, ^60^, ^61^, ^62^ ^]^ ecology and evolutionary biology,^[^ ^63^, ^64^, ^65^ ^]^ social dynamics,^[^ ^66^, ^67^, ^68^, ^69^, ^70^ ^]^ and interdisciplinary work.^[^ ^71^ ^]^

In this paper, we propose a compartmental population model, which we call the SIADE model, that takes into account the health and well‐being of the population, as well as economic impacts. In this SIADE model, we partition the entire population of a community into five different compartments. The general population is susceptible (*S*). During the epidemic, people in *S* can be isolated (*I*). The people in *S* can also be infected, and an infected patient is either ailing (*A*) or diagnosed (*D*). Finally, the portion of population that succumbs to the disease is counted toward the extinct population (*E*). By using the SIADE model, it provides a framework to calculate the “cost” borne by each response strategy to COVID‐19 management (which is strictly based on the size of each population compartment). The employed strategy will determine how the population flows in each compartment. Meanwhile, a cost function that takes into consideration the health and well‐being of the population, as well as economic impact, is proposed to compute the cost attributed to the different strategies. This index will indicate the effectiveness of the different suppression strategies and reveal how two losing strategies can be combined to achieve a winning outcome. Section 2 shows the population interaction model and presents the results of the various strategies employed. Subsequently, we provide analysis and discussion in Section 3. We further develop the methods in Section 4. More details on these parameter functions can be found in the Supporting Information.

## 2 Results

### 2.1 Population Model

**Figure 1:**
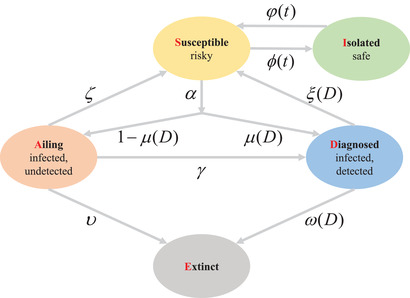
The SIADE model depicting the interaction between each compartment. The population is partitioned into five compartments: Susceptible (S), Isolated (I), Ailing (A), Diagnosed (D), and Extinct (E), respectively.

The SIADE model describes the interaction and flow between the different population compartments during the COVID‐19 epidemic. In this model, there are five different compartments. During an epidemic, the government can impose a strict lockdown strategy, which shifts the susceptible population from *S* into isolation *I* at a rate *ϕ*(*t*). Similarly, an isolated population *I* can come out of isolation and become susceptible *S* at rate φ(*t*). The susceptible population *S* can be infected at rate α. However, after infection, only a portion is detected and diagnosed *D*, with probability μ(*D*). The remaining infected population that goes undetected is classified as ailing *A*. The isolated population is assumed to be effective in preventing further infection,^[^ ^72^ ^]^ in that the isolated population will not be infected. With detecting, the ailing population can be diagnosed. This is proportional to the detection rate γ. Individuals in both the ailing (*A*) and diagnosed (*D*) populations can recover with rates ζ and ξ(*D*), respectively, or die with rates υ and ω(*D*), respectively. The evolution of the population in different stages over time, is shown below:

(1) $$\begin{array}{ccc} \dot{S}\left(t\right) & = & -\alpha S(t)A(t)-\phi (t)S(t)+\varphi (t)I(t)+\xi (D)D(t)+\zeta A(t)\\ \dot{I}\left(t\right) & = & \phi (t)S(t)-\varphi (t)I(t)\\ \dot{A}\left(t\right) & = & \alpha \left(1-\mu (D)\right)S\left(t\right)A\left(t\right)-\gamma A\left(t\right)-\upsilon A\left(t\right)-\zeta A\left(t\right)\\ \dot{D}\left(t\right) & = & \alpha \mu (D)S(t)A(t)+\gamma A(t)-\omega (D)D(t)-\xi (D)D(t)\\ \dot{E}\left(t\right) & = & \upsilon A(t)+\omega (D)D(t) \end{array}$$

where *S*(*t*), *I*(*t*), *A*(*t*), *D*(*t*), *E*(*t*) refer to the population sizes of each compartment at any given time *t*. In this model, we assume no new birth or natural death, thus, *S*(*t*) + *I*(*t*) + *A*(*t*) + *D*(*t*) + *E*(*t*) is a constant for all *t*. **Figure** 1 illustrates the interactions among different populations. The initial values of the different populations and flow rates used in this paper are shown in **Table** 1. The parameter functions in Table 1 are further discussed in the Supporting Information.

**Table 1:** Description of initial conditions and parameters

Parameter	Description	Initial conditions
*S*(0)	Number of susceptible people	10^7^
*I*(0)	Number of isolated people	200
*A*(0)	Number of ailing people	200
*D*(0)	Number of diagnosed people	0
*E*(0)	Number of extinct people	0
α	Infection rate	1.4 × 10^−7^
ζ	Recovery rate of *A*	0.2
υ	Mortality rate of *A*	0.5 × 10^−2^
γ	Detection rate	Random [0.1, 0.2]
*ϕ*(*t*)	Isolation rate	0
φ(*t*)	Discontinue isolation rate	0
ξ(*D*)	Recovery rate of *D*	*S*_*d*_(*D*, 2.5 × 10^4^, 3 × 10^3^, 0.5, 0.45)
μ(*D*)	Probability of diagnosis on infection	*S*_*u*_(*D*, 2 × 10^4^, 7 × 10^3^, 0.5, 0.45)
ω(*D*)	Mortality rate of *D*	*S*_*u*_(*D*, 3 × 10^4^, 3 × 10^3^, 0, 10^−3^)

### 2.2 Strategies Implemented

In an epidemic, the government can employ several strategies to combat the societal implications of the disease. In this context, we define a losing strategy as one bearing a substantial cost to the society.

#### 2.2.1 Individual Strategies

In our case, we consider a) open community and b) lockdown individually. The details of both strategies are discussed below.

a. **Open community (Strategy A)** will not encourage isolation. This will see a flow of population from *I* to *S*, if the population is initially in isolation. The implementation is as follows:
  1) φ(*t*) = *S*_*u*_(*t* − *t*_*start*_, 10, 7, 0.05, 0.05) + *r*, where *r* is a random number drawn uniformly from [ − 0.03, 0.03].
b. **Lockdown (Strategy B)** imposes restrictions on the physical movement of the population in *S*, resulting in *S* mass converting to *I*. This will also facilitate an improvement in the detection rate γ. We also assume that individuals who feel unwell will go to the hospital at the soonest available time, resulting in an improvement to μ(*D*). Notably, in our model, we impose an upper limit to *I*, set at 9.5 × 10^6^. This accounts for workers required to maintain essential activities such as food supply, transportation, and daily operations in the hospitals when most people are isolated and infected. The implementation is as follows:
  1. *ϕ*(*t*) = *S*_*u*_(*t* − *t*_*start*_, 20, 10, 0.05, 0.05) + *r*, where *r* is a random number drawn uniformly from [ − 0.03, 0.03];
  2. γ increases from its previous value to a random number in [0.2, 0.3];
  3. μ(*D*) is *S*_*u*_(*D*, 10^4^, 5 × 10^3^, 0.5, 0.45).
  The random number *r* in φ(*t*) and *ϕ*(*t*) is incorporated in both strategies to account for a small portion of the population that does not adhere to the prescribed strategy.

#### 2.2.2 Alternating Strategies

There are three different types of alternating strategies to be studied in this paper. These strategies employ various switching rules involving both of the individual strategies A and B. They are the a) time‐based switching scheme, b) result‐based switching scheme, and c) random switching scheme.

a. **Time‐based switching scheme** alternates between strategy A and strategy B based solely on time *t*,^[^ ^73^ ^]^ that is, if
  (2) $$\left\{\begin{array}{c} 0\leq \;\mathrm{mod}\;\left(t,T\right)<t_{1},\;\text{employ}\;\text{strategy}\;A\\ t_{1}\leq \;\mathrm{mod}\;\left(t,T\right)<T,\;\text{employ}\;\text{strategy}\;B \end{array}\right.$$
  This means that strategy A and strategy B will be applied in sequence. Open community is implemented in the period [0, *t*_1_) and lockdown is imposed in the period [*t*_1_, *T*). For illustrative purpose, we use *T* = 10, *t*_1_ = 5 in the numerical experiments in this paper.
b. **Result‐based switching scheme** has dynamics similar to the time‐based switching scheme. However, instead of switching according to time, this switching scheme is decided by the number of new infections from the previous day, that is, if
  (3) $$\left\{\begin{array}{c} \alpha S\left(t\right)A\left(t\right)<L_{D},\;\text{employ}\;\text{strategy}\;A\\ \alpha S\left(t\right)A\left(t\right)>L_{U},\;\text{employ}\;\text{strategy}\;B \end{array}\right.$$
  Here, α*S*(*t*)*A*(*t*) is the number of new infections per day. When the infection count is higher than *L*_*U*_ = 6000, lockdown is imposed. On the contrary, an open community strategy is then applied when the infection count is lower than *L*_*D*_ = 1000. When α*S*(*t*)*A*(*t*) is in [*L*_*D*_, *L*_*U*_], the community will continue with the strategy from the previous day.
c. **Random switching scheme** will adjust the implementation strategy arbitrarily every day. In the long term, each strategy will be implemented with probability 0.5.

### 2.3 Cost Function

We quantify the efficacy of each strategy via a cost function. The cost function takes into account the loss to both society and individuals in the form of hospitalization cost, personal opportunity cost, human capital investment (defined as the economic value of a worker's experience and skills), and the cost of risky behavior.^[^ ^74^ ^]^ The cost function *F*(*t*) is defined as

(4) $$F\left(t\right)=F_{H}\left(t\right)+F_{I}\left(t\right)+F_{C}\left(t\right)+F_{R}\left(t\right)$$

where *F*_*H*_(*t*), *F*_*I*_(*t*), *F*_*C*_(*t*), and *F*_*R*_(*t*) are the hospitalization cost, individual opportunity cost due to isolation, human capital investment, and the cost of risky behavior, respectively. The way we quantify each term can be found in the Supporting Information. Meanwhile, the cumulative cost F(t) is considered together with the “cost” per day. The cumulative cost F(t) is the cumulative cost from the beginning to time *t*. More details can be found in the Supporting Information.

### 2.4 Rising Cost Arising from Individual Strategies

**Figure 2:**
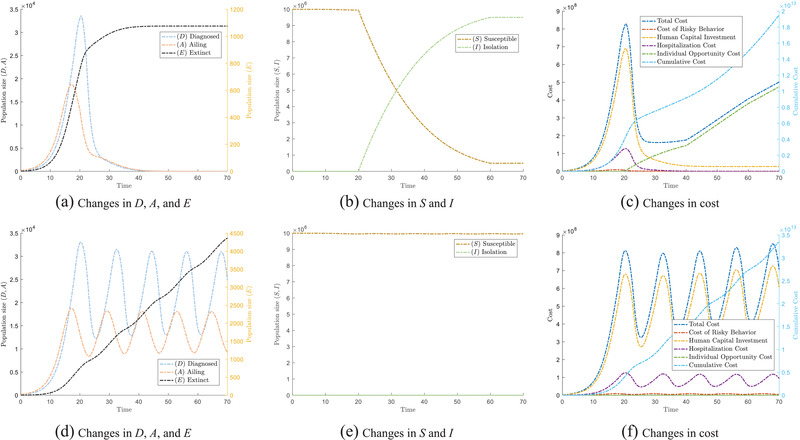
The epidemic will have different trends under different single strategies. (a–c) show the trend under lockdown, while (d)–(f) show the trend for an open community strategy. In particular, (a) and (d) show the changes of population which are affected by COVID, that is, Ailing (A), Diagnosed (D), and Extinct (E); (b) and (e) show the changes of Susceptible (S) and Isolated (I); (c) and (f) show the change in cost under different single strategies.

As noted in our description of this model, neither strategy A nor strategy B can individually result in a decline to the “cost” during an epidemic. **Figure** 2a–c shows the evolution and flow of populations in each compartment and the “cost” under lockdown. Figure 2d–f depicts the same for an open community. Clearly, our simulation results show that either individual strategy is a losing one.

Under the lockdown strategy (Figure 2a), we observe that *D* and *A* have reduced drastically after the implementation of a lockdown. As the population remains in lockdown after the first wave of epidemic around *t* = 25, this aggressive strategy results in the eradication of the number of people infected by the virus around *t* = 40. These simulation results show the effectiveness of a lockdown in response to epidemics that spread through human contact. Figure 2b shows that as more people go into isolation, the virus spreads less easily. However, effective as this strategy is, the total cost starts to rise when the lockdown is imposed, as observed in Figure 2c. The cost then further increases at a faster rate beyond *t* = 40. The initial decline is attributed to decreases in the loss of human capital *F*_*C*_(*t*) and in hospitalization cost *F*_*H*_(*t*). Meanwhile, *F*_*C*_(*t*) and *F*_*H*_(*t*) are the direct outcome of the number of individuals in *E*(*t*) and *D*(*t*). However, this is soon outweighed by the individual opportunity cost due to isolation. *F*_*I*_(*t*) rises faster than the decline of other costs, when the isolation period reaches *T*_*I*_ (See Supporting Information for details on *T*_*I*_). Hence, the total cost *F*(*t*) will continue to increase after *t* = 40. This rising cost allows us to classify strategy B as a losing strategy. The same analysis applies to strategy A, leading to the same conclusion. Of particular interest is the smooth “ratcheting” behavior of the cost. Such phenomenon is reminiscent of Parrondo's paradox.^[^ ^30^ ^]^ The cumulative cost F(t) of individual strategy A and strategy B tracks the accumulated cost due to the epidemic. (See Supporting Information for a definition of cumulative cost.) We will now exploit the ratcheting effect to our advantage by considering the switching between both strategies.

### 2.5 Controlled Loss under Alternating Strategies

**Figure 3:**
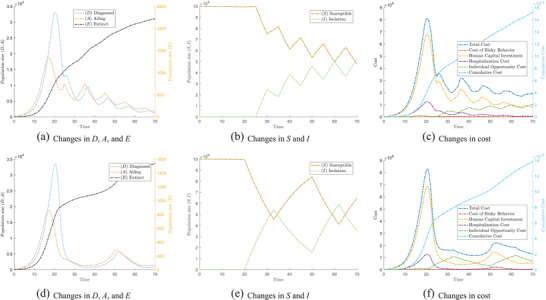
The epidemic evolves differently under different switching strategies. (a)–(c) show the tendency under the time‐based switching scheme. (d)–(f) show the tendency under result‐based switching scheme. In particular, (a) and (d) show the changes of population which are affected by COVID‐19, that is, Ailing (A), Diagnosed (D), and Extinct (E); (b) and (e) show the changes of Susceptible (S) and Isolated (I); (c) and (f) show the change in cost under different switching strategies.

Despite both strategies (lockdown and open community) individually resulting in “losing” outcomes as defined in the previous subsection, we show here that it is possible to obtain a “winning” outcome by switching between the two strategies. Here, a “winning” outcome is defined as a strategy that results in a moderated cost, while not compromising the number of individuals in *E*. A winning outcome can be achieved because switching from strategy B to strategy A allows the population in *I* to flow to *S*. As a result, this reduces the cost due to *F*_*I*_(*t*), which is caused by an extended period of isolation. At the same time, switching from strategy A to strategy B significantly reduces the hospitalization cost *F*_*H*_(*t*) and human capital investment *F*_*C*_(*t*) resulting from infections. Our simulation results show that both the time‐based switching and result‐based switching scheme can control the “cost” caused by an epidemic. **Figure** 3a–c shows the results due to the time‐based switching scheme. Figure 3d‐f shows the results derived from the result‐based switching scheme.

When alternating between the two strategies according to the time‐based switching scheme, we observe that the rate of increase in population *E* decreases, and remains lower than if we were to employ strategy B individually. Meanwhile, the net population sizes of *D* and *A* gradually decrease in each instance of the switching. This proves that the switching strategy is useful in controlling the epidemic. We observe a downward trend for both populations *A* and *D*. Closer inspection of the population size of *S* and *I* in Figure 3b shows that time‐based switching gradually increases the population in isolation *I*. This ratcheting effect means that no one single cost can significantly outweigh the three other cost components, and thus the cost decreases and stabilizes over time as observed in Figure 3c. As the strategies are time‐based, the periodic rise and fall in the number of infected individuals will be largely predictable and periodic in real life. A similar trend can be observed from the result‐based switching scheme. It is worth noting that, as result‐based switching is based on the number of infections from the previous day, it employs a stricter condition for switching between strategy A and strategy B. This allows populations *A* and *D* to fall significantly, before a second wave is observed again, as depicted in Figure 3d. This stricter control measure also results in fewer deaths. Our simulation results (not shown here) also revealed that even the random switching scheme based on a simple coin toss can result in relieving the “cost” borne during an epidemic when compared against the cost of sticking to an individual strategy. This shows that our model is robust across a wide range of switching strategies. However, the random switching scheme may be difficult to implement in the real world. It will not be further discussed in this paper.

### 2.6 Sensitivity Analysis Test

**Figure 4:**
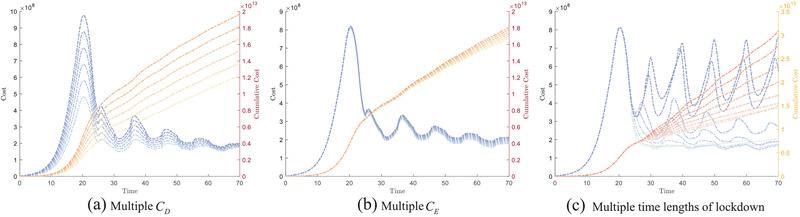
The variation in cost function *F*(*t*) and cumulative cost F(t) under sensitivity analysis of the parameters. (a) shows *F*(*t*) and F(t) as a function of *C*_*D*_ ∈ [10000, 25000] in intervals of 3000. (b) shows *F*(*t*) and F(t) as a function of *C*_*E*_ ∈ [20000, 35000] in intervals of 3000. (c) shows the variation in lockdown duration by varying *t*_1_ ∈ [1, 9] in intervals of 1 in the time‐based switching scheme (*T* = 10).

We want to study the effects of different parameters on the total cost *F*(*t*) and cumulative cost F(t). Firstly, when implementing the time‐based switching scheme, we studied the impact of changing *C*_*D*_ and *C*_*E*_ on the cost *F*(*t*) and F(t). The various outcomes of *F*(*t*) and F(t) under different values of *C*_*D*_ and *C*_*E*_ are shown in **Figure** 4a and Figure 4b, respectively. In our simulations, we consider *C*_*D*_ ∈ [10000, 25000] in intervals of 3000 and *C*_*E*_ ∈ [20000, 35000] in intervals of 3000. A darker color of the line represents a greater *C*_*D*_ and *C*_*E*_. From Figure 4a, we can conclude that a change in *C*_*D*_ causes a large change in *F*(*t*) in the early stage ([10, 30]), because *C*_*D*_ mainly depends on the number of individuals in population *D*. As discussed in the previous subsection, since the alternating strategy is only implemented at *t* = 20, the population count in *D* is still high. The difference of *F*(*t*) in the later stage is small, because the epidemic is well controlled at that stage. Thus we do not expect to see large variations in *F*(*t*) despite changing weight *C*_*D*_. Contrary to *C*_*D*_, the differences in *F*(*t*) due to varying *C*_*E*_ is mainly observed in the later period. This is because, as the epidemic develops, the number in *E* gradually increases, resulting in greater variations in *F*(*t*).

Next, we study the impact of the lockdown duration on the cost function *F*(*t*) and cumulative cost F(t) using the time‐based switching scheme in Figure 4c. In this numerical experiment, we fixed the cycle period *T* = 10, with *t*_1_ ∈ [1, 9] in intervals of 1 (i.e., for smaller *t*_1_, the population is in lockdown for the majority of the cycle period). A darker color of the line represents a greater *t*_1_. The observed trend confirms the intuition that in the extreme cases, regardless of whether *t*_1_ is small or large, we do not obtain a decrease in the cost function. This is attributed to the fact that in these extreme cases, the time‐based switching strategy is very similar to the individual strategy, and so the cost function *F*(*t*) also displays the same outcome.

The cumulative cost F(t) trend in each figure is based on the value of *F*(*t*). F(t) will increase more rapidly with larger *F*(*t*) in each day, as observed in Figure 4. We also observe that we will obtain a higher F(t) with higher *C*_*D*_, *C*_*E*_, and *t*_1_ values. It is worth noting that when *t*_1_ reaches its lower bound, F(t) does not change significantly (as opposed to at the upper limit of *t*_1_). This is because *F*(*t*) has a smaller variation in the later period.

## 3 Discussion

In this study, we have shown the paradoxical result of switching between two losing strategies to lower the “cost” of an epidemic via Parrondo's games. We have also quantified and analysed the impact of different strategies on the health and well‐being of the population, as well as on the economy. When combining two losing strategies, we show that one can achieve a winning result, defined as a strategy that leads to a decline in the “cost” per day. From this study, we show that keeping the community open results in a large number of infected individuals and a sharp increase in the number of deaths over time, so naturally the “cost” also increases. At the same time, a lockdown strategy reduces the possibility of infection, but has an adverse effect on the socio‐economic cost. This means that neither strategy A nor strategy B can individually result in a decline to the “cost” in the long‐term during an epidemic. Such rising “cost” allows us to classify them as losing strategies. Meanwhile, if most people do not comply with the compulsory lockdown strategy, or if strategy is not maintained long enough, the benefits will be significantly reduced. Our proposed model also shows that alternating strategies will gradually bring down the costs.

Our model is important as it reveals the possible strategies that can be implemented to curb the spread of COVID‐19 or future epidemics. When one switches between the losing strategies in accordance with any of the proposed alternating strategies, the “cost” per day will decline. This, in itself, is a winning strategy to control the loss caused by COVID‐19. In this paper, three different switching rules have been introduced. They are the time‐based switching, result‐based switching, and random switching scheme. While the first two switching schemes can be evaluated and realistically implemented in the real‐world, the random switching scheme may not be feasible due to the potential confusion that it may bring about to the residents.

Regarding the switching strategies, the result‐based switching scheme sees a steeper fall in the total “cost” compared to the time‐based switching scheme. The increasing trend of F(t) in these two alternating strategies is slower than that for either individual strategy (deployed on its own). Our results have shown that an alternating strategy following any of the switching rules will effectively reduce the “cost” caused by an epidemic, a counter‐intuitive result exploited through Parrondo's games. It is important to point out that regardless of which alternating strategy is adopted, the resulting total cost *F*(*t*) is about an order of magnitude lower than that of the individual strategy implementation. Finally, we conduct a sensitivity analysis test by varying the weights involved in populations *D* and *E*. We conclude that the parameter *C*_*D*_ associated with population *D* affects the variation of cost in the early stage of strategy implementation, while *C*_*E*_ affects the later stage. We expect these results to serve as simple but strong baselines that can motivate future work in lockdown exit strategies. This work is useful for policy makers to chart a strategy to manage the epidemic in their respective country. In our model, we have considered the health and well‐being of the population, as well as the economic impact. In order for the model to remain tractable, there are other costs and gains which are not considered here. We further note that changes in the characteristics of the strain of the virus, like the severe disease rate and infectivity, caused by mutations are not considered in this model. The novelty of our work lies in designing a methodology and mathematical framework to combine two losing strategies in a compartmental population model to achieve a winning outcome—a phenomenon attributed to Parrondo's paradox.

## 4 Experimental Section

MATLAB 2016a with *ode23* differential equation (ODE) solver was used to simulate the population model. Simulations under different parameters showed that the lockdown strategy and open community strategy are both losing strategies in the long‐term, resulting in the greatest cost to society as quantified by the cost function. The initial value of different populations and parameters are shown in Table 1. The population starts as an open community at time *t* = 0 and strategies will be implemented according to the rules described above. The period of open community is set to be from *t* = [0, 20], that is, the first instance at which a decision needs to be made (on which strategy to employ) is *t* = 20^+^. Under the switching strategy in Equations (2) and (3), the strategy will be switched only once a day, rather than detecting time *t* and number of new infections α*S*(*t*)*A*(*t*) at any time. When the detected data meets the switching conditions, the parameters will be modified to pre‐defined values in the switching rule. Then, the population interaction model will continue to be simulated under new parameter conditions. The cost at any time, which considers the health and well‐being of the population, as well as the economy, will be calculated by Equation (4) to evaluate the efficacy of each strategy. Published real‐world data from organization and government report (available in refs. 72, 75, 76, 77) were used to validate the model with parameter values given in the Supporting Information. The parameter functions^78^ in Table 1 are further discussed in the Supporting Information.

## Conflict of Interest

The authors declare no conflict of interest.

## Supporting information

Supporting Information

## References

[1] C. Huang , Y. Wang , X. Li , L. Ren , J. Zhao , Y. Hu , L. Zhang , G. Fan , J. Xu , X. Gu , Z. Cheng , T. Yu , J. Xia , Y. Wei , W. Wu , X. Xie , W. Yin , H. Li , M. Liu , Y. Xiao , H. Gao , L. Guo , J. Xie , G. Wang , R. Jiang , Z. Gao , Q. Jin , J. Wang , B. Cao , Lancet 2020, 395, 497 31986264 10.1016/S0140-6736(20)30183-5 PMC7159299

[2] D. Wang , B. Hu , C. Hu , F. Zhu , X. Liu , J. Zhang , B. Wang , H. Xiang , Z. Cheng , Y. Xiong , Y. Zhao , Y. Li , X. Wang , Z. Peng , JAMA 2020, 323, 1061. 32031570 10.1001/jama.2020.1585 PMC7042881

[3] W.‐j. Guan , Z.‐y. Ni , Y. Hu , W.‐h. Liang , C.‐q. Ou , J.‐x. He , L. Liu , H. Shan , C.‐l. Lei , D. S. Hui , B. Du , L.‐j. Li , G. Zeng , K.‐Y. Yuen , R.‐c. Chen , C.‐l. Tang , T. Wang , P.‐y. Chen , J. Xiang , S.‐y. Li , J.‐l. Wang , Z.‐j. Liang , Y.‐x. Peng , L. Wei , Y. Liu , Y.‐h. Hu , P. Peng , J.‐m. Wang , J.‐y. Liu , Z. Chen , et al., N. Engl. J. Med. 2020, 382, 1708. 32109013 10.1056/NEJMoa2002032 PMC7092819

[4] Johns Hopkins University, Coronavirus resource center, https://coronavirus.jhu.edu/map. Accessed June 20, 2020.

[5] A. Alimadadi , S. Aryal , I. Manandhar , P. B. Munroe , B. Joe , X. Cheng , Physiol. Genomics 2020, 52, 200. 32216577 10.1152/physiolgenomics.00029.2020 PMC7191426

[6] C. Rajivganthi , F. A. Rihan , S. Lakshmanan , R. Rakkiyappan , P. Muthukumar , Complexity 2016, 21, 412.

[7] L. Yan , H.‐T. Zhang , J. Goncalves , Y. Xiao , M. Wang , Y. Guo , C. Sun , X. Tang , L. Jing , M. Zhang , X. Huang , Y. Xiao , H. Cao , Y. Chen , T. Ren , F. Wang , Y. Xiao , S. Huang , X. Tan , N. Huang , B. Jiao , C. Cheng , Y. Zhang , A. Luo , L. Mombaerts , J. Jin , Z. Cao , S. Li , H. Xu , Y. Yuan , Nat. Mach. Intell. 2020, 2, 283.

[8] R. Saravanakumar , H. Mukaidani , P. Muthukumar , Neurocomputing 2020, 406, 244.

[9] G. Giordano , F. Blanchini , R. Bruno , P. Colaneri , A. Di Filippo , A. Di Matteo , M. Colaneri , Nat. Med. 2020, 26, 855. 32322102 10.1038/s41591-020-0883-7 PMC7175834

[10] B. Wei , F. Xiao , Y. Shi , IEEE Trans. Cybern. 2020, 50, 2926. 31634858 10.1109/TCYB.2019.2944971

[11] B. Wei , F. Xiao , Y. Shi , IEEE Trans. Cybern. 2020, 50, 2380. 31581106 10.1109/TCYB.2019.2940987

[12] D. M. Gysi , Í. D. Valle , M. Zitnik , A. Ameli , X. Gan , O. Varol , H. Sanchez , R. M. Baron , D. Ghiassian , J. Loscalzo , A.‐L. Barabási , *arXiv 2004.07229* , 2020.

[13] S. Sheykhali , A. H. Darooneh , G. R. Jafari , Phys. A 2019, 548, 123882.

[14] M. Bahrami , N. Chinichian , A. Hosseiny , G. Jafari , M. Ausloos , Phys. A 2020, 540, 123203.

[15] M. Lazzerini , G. Putoto , Lancet Glob. Health 2020, 8, e641. 32199072 10.1016/S2214-109X(20)30110-8 PMC7104294

[16] X. Deng , W. Jiang , Int. J. Intell. Syst. 2019, 34, 3302.

[17] X. Deng , Int. J. Intell. Syst. 2018, 33, 1869.

[18] N. Chen , M. Zhou , X. Dong , J. Qu , F. Gong , Y. Han , Y. Qiu , J. Wang , Y. Liu , Y. Wei , J. Xia , T. Yu , X. Zhang , L. Zhang , The Lancet 2020, 395, 507. 10.1016/S0140-6736(20)30211-7 PMC7135076 32007143

[19] S. M. Kissler , C. Tedijanto , E. Goldstein , Y. H. Grad , M. Lipsitch , Science 2020, 368, 860. 32291278 10.1126/science.abb5793 PMC7164482

[20] R. Li , S. Pei , B. Chen , Y. Song , T. Zhang , W. Yang , J. Shaman , Science 2020, 368, 489. 32179701 10.1126/science.abb3221 PMC7164387

[21] N. Zhu , D. Zhang , W. Wang , X. Li , B. Yang , J. Song , X. Zhao , B. Huang , W. Shi , R. Lu , P. Niu , F. Zhan , X. Ma , D. Wang , W. Xu , G. Wu , G. F. Gao , W. Tan , N. Engl. J. Med. 2020, 382, 727. 31978945 10.1056/NEJMoa2001017 PMC7092803

[22] K. H. Cheong , M. C. Jones , Bioessays 2020, 42, 2000063.

[23] P. Block , M. Hoffman , I. J. Raabe , J. B. Dowd , C. Rahal , R. Kashyap , M. C. Mills , Nat. Hum. Behav. 2020, 1. 32499576 10.1038/s41562-020-0898-6

[24] H. Lau , V. Khosrawipour , P. Kocbach , A. Mikolajczyk , J. Schubert , J. Bania , T. Khosrawipour , J. Travel Med. 2020, 27. 10.1093/jtm/taaa037 PMC7184469 32181488

[25] F. Alvarez , D. Argente , F. Lippi , National Bureau of Economic Research 2020, 10, w26981.

[26] D. Guan , D. Wang , S. Hallegatte , S. J. Davis , J. Huo , S. Li , Y. Bai , T. Lei , Q. Xue , D. Coffman , D. Cheng , P. Chen , X. Liang , B. Xu , X. Lu , S. Wang , K. Hubacek , P. Gong , Nat. Hum. Behav. 2020, 1–11. 10.1038/s41562-020-0896-8 32493967

[27] C.‐C. Lee , W.‐C. Poon , J. Contemp. Account. Econ. 2018, 14, 335.

[28] P. G. Walker , C. Whittaker , O. J. Watson , M. Baguelin , P. Winskill , A. Hamlet , B. A. Djafaara , Z. Cucunubá , D. O. Mesa , W. Green , H. Thompson , S. Nayagam , K. E. C. Ainslie , S. Bhatia , S. Bhatt , A. Boonyasiri , O. Boyd , N. F. Brazeau , L. Cattarino , G. Cuomo‐Dannenburg , A. Dighe , C. A. Donnelly , I. Dorigatti , S. L. van Elsland , R. F. John , H. Fu , K. A. M. Gaythorpe , L. Geidelberg , N. Grassly , D. Haw , et al., Science 2020, 369, 413. 32532802 10.1126/science.abc0035 PMC7292504

[29] L. L. Chuah , W. C. Poon , B. K. Guru , Mod. Appl. Sci. 2018, 12, 9.

[30] G. P. Harmer , D. Abbott , Nature 1999, 402, 864.

[31] J. Rousselet , L. Salome , A. Ajdari , J. Prostt , Nature 1994, 370, 446. 8047163 10.1038/370446a0

[32] J. M. Parrondo , G. P. Harmer , D. Abbott , Phys. Rev. Lett. 2000, 85, 5226. 11102227 10.1103/PhysRevLett.85.5226

[33] R. Toral , P. Amengual , S. Mangioni , Phys. A 2003, 327, 105.

[34] K. H. Cheong , J. M. Koh , Ultramicroscopy 2019, 202, 100. 31005022 10.1016/j.ultramic.2019.03.004

[35] J. M. Koh , K. H. Cheong , J. Electron Spectrosc. Relat. Phenom. 2018, 227, 31.

[36] A. Rosato , K. J. Strandburg , F. Prinz , R. H. Swendsen , Phys. Rev. Lett. 1987, 58, 1038. 10034316 10.1103/PhysRevLett.58.1038

[37] R. Pinsky , M. Scheutzow , Annales de l'I.H.P. Probabilités et statistiques 1992, 28, 519.

[38] C. E. M. Pearce , AIP Conf. Proc. 2000, 511, 207.

[39] C. E. M. Pearce , AIP Conference Proceedings 2000, 511, 207.

[40] K. H. Cheong , D. B. Saakian , R. Zadourian , Phys. Rev. E 2017, 96, 062303. 29347428 10.1103/PhysRevE.96.062303

[41] J. Almeida , D. Peralta‐Salas , M. Romera , Phys. D 2005, 200, 124.

[42] L. Kocarev , Z. Tasev , Phys. Rev. E 2002, 65, 046215. 10.1103/PhysRevE.65.046215 12005984

[43] M.‐F. Danca , M. Fečkan , M. Romera , Int. J. Bifurcation Chaos 2014, 24, 1450008.

[44] M.‐F. Danca , W. K. Tang , Chin. Phys. B 2015, 25, 010505.

[45] M.‐F. Danca , J. Chattopadhyay , Chaos 2016, 26, 043106. 27131485 10.1063/1.4946811

[46] J. S. Cánovas , Phys. A 2017, 466, 153.

[47] S. A. Mendoza , E. W. Matt , D. R. Guimarães‐Blandón , E. Peacock‐López , Chaos, Soliton. Fract. 2018, 106, 86.

[48] J. Buceta , K. Lindenberg , J. M. Parrondo , Phys. Rev. Lett. 2001, 88, 024103. 11801018 10.1103/PhysRevLett.88.024103

[49] M.‐F. Danca , Commun. Nonlinear Sci. Numer. Simul. 2013, 18, 500.

[50] S. Jia , J. W. Lai , J. M. Koh , N. G. Xie , K. H. Cheong , Phys. A 2020, 556, 124714.

[51] D. A. Meyer , H. Blumer , J. Stat. Phys. 2002, 107, 225.

[52] D. A. Meyer , H. Blumer , Fluctuation Noise Lett. 2002, 2, L257.

[53] C. F. Lee , N. F. Johnson , F. Rodriguez , L. Quiroga , Fluctuation Noise Lett. 2002, 2, L293.

[54] J. Rajendran , C. Benjamin , R. Soc. Open Sci. 2018, 5, 171599. 29515873 10.1098/rsos.171599 PMC5830762

[55] J. Rajendran , C. Benjamin , Europhys. Lett. 2018, 122, 40004.

[56] J. W. Lai , K. H. Cheong , Nonlinear Dyn. 2020, 100, 849.

[57] J. W. Lai , K. H. Cheong , Phys. Rev. E 2020, 101, 052212. 32575251 10.1103/PhysRevE.101.052212

[58] K. H. Cheong , J. M. Koh , M. C. Jones , BioEssays 2019, 41, 1900027.

[59] F. A. Reed , Genetics 2007, 176, 1923. 17483431 10.1534/genetics.106.069997 PMC1931524

[60] K. H. Cheong , J. M. Koh , M. C. Jones , Proc. Natl. Acad. Sci. 2018, 115, E5258. 29752380 10.1073/pnas.1806485115 PMC6003326

[61] K. H. Cheong , J. M. Koh , M. C. Jones , Fluctuation Noise Lett. 2019, 18, 1971001.

[62] J. M. Koh , N.‐g. Xie , K. H. Cheong , Nonlinear Dyn. 2018, 94, 1467.

[63] Z.‐X. Tan , K. H. Cheong , Nonlinear Dyn. 2019, 98, 1.

[64] P. D. Williams , A. Hastings , Proc. R. Soc. B 2011, 278, 1281. 10.1098/rspb.2010.2074 PMC3061147 21270032

[65] Z.‐X. Tan , J. M. Koh , E. V. Koonin , K. H. Cheong , Adv. Sci. 2020, 7, 1901559. 10.1002/advs.201901559 PMC7001654 32042555

[66] H. F. Ma , K. W. Cheung , G. C. Lui , D. Wu , K. Y. Szeto , Comput. Econ. 2019, 54, 1491.

[67] Y. Ye , X. R. Hang , J. M. Koh , J. A. Miszczak , K. H. Cheong , N. G. Xie , Nonlinear Dyn. 2019, 98, 1821.

[68] Y. Ye , X. R. Hang , J. M. Koh , J. A. Miszczak , K. H. Cheong , N.‐g. Xie , Chaos Soliton. Fract. 2020, 130, 109464.

[69] J. M. Koh , K. H. Cheong , Nonlinear Dyn. 2019, 96, 257.

[70] J. M. Koh , K. H. Cheong , Nonlinear Dyn. 2019, 98, 943.

[71] J. W. Lai , J. Chang , L. K. Ang , K. H. Cheong , Inform. Fus. 2020, 63, 248.

[72] J. Hellewell , S. Abbott , A. Gimma , N. I. Bosse , C. I. Jarvis , T. W. Russell , J. D. Munday , A. J. Kucharski , W. J. Edmunds , F. Sun , S. Flasche , B. J. Quilty , N. Davies , Y. Liu , S. Clifford , P. Klepac , M. Jit , C. Diamond , H. Gibbs , K. van Zandvoort , Lancet Glob. Health 2020. 10.1016/S2214-109X(20)30074-7 PMC7097845 32119825

[73] K. H. Cheong , Z. X. Tan , Y. H. Ling , Commun. Nonlinear Sci. Numer. Simulat. 2018, 60, 107.

[74] R. Laxminarayan , A. Malani , in The Oxford Handbook of Health Economics (Eds: Glied S. and Smith P. C.), Oxford University Press, Oxford 2011.

[75] WHO report, https://www.who.int/docs/default-source/coronaviruse/situation-reports/20200306-sitrep-46-covid-19.pdf?sfvrsn=96b04adf_4. Accessed June 20, 2020.

[76] National healthcare security administration of the People's Republic of China. http://www.gov.cn/xinwen/2020-04/12/content_5501508.htm. Accessed June 20, 2020.

[77] 116th United States Congress. Coronavirus aid, relief, and economic security act, 2020 https://www.congress.gov/bill/116th-congress/house-bill/748/. Accessed June 20, 2020.

[78] M. Kuwamura , T. Nakazawa , T. Ogawa , J. Math. Biol. 2009, 58, 459. 18663449 10.1007/s00285-008-0203-1

