# Supplementary material for: Relieving Cost of Epidemic by Parrondo's Paradox: A COVID‐19 Case Study
Source: Adv Sci (Weinh). 2020 Nov 5;7(24):2002324. doi: 10.1002/advs.202002324 (PMC7740105; doi:10.1002/advs.202002324)
Supplement: Supplementary file 1 — Supporting Information [file ADVS-7-2002324-s001.pdf]

© 2020 Wiley-VCH GmbH

ADVANCED SCIENCE
Open Access

Supporting Information

for *Adv. Sci.*, DOI: 10.1002/advs. 202002324

Relieving cost of epidemic by Parrondo's paradox: A COVID-19 case study

Kang Hao Cheong, Tao Wen, Joel Weijia Lai*

Supporting Information

Relieving cost of epidemic by Parrondo's paradox: A COVID-19 case study

Kang Hao Cheong[1,*], Tao Wen[1], and Joel Weijia Lai[1]

[1]Science, Mathematics and Technology Cluster, Singapore University of Technology and Design, 8 Somapah Rd, S487372, Singapore

[*]Corresponding author: Kang Hao Cheong (kanghao_cheong@sutd.edu.sg)

Determining Parameter Functions $\xi(D)$, $\mu(D)$ and $\omega(D)$

To determine how the flow rate parameters switch with the employed strategy, we make use of the sigmoid function[1] to determine the switching threshold. There are two sigmoid switching functions defined as follows:

$$S_u(x) = \frac{1}{1+\exp(-\frac{x-\eta}{\sigma})},$$
$$S_d(x) = \frac{1}{1+\exp(\frac{x-\eta}{\sigma})}.$$

η is the switching threshold where $S_u(\eta) = 1/2$ and $S_d(\eta) = 1/2$ for some $x = \eta$. σ is a tolerance parameter that controls the sharpness of switching. That is, the smaller the σ, the steeper the sigmoid function in the region of tolerance, thus affecting the tendency of switching from one strategy to another.

The standard sigmoid function has a domain $[0, 1]$. However, the parameters used in our model do not necessarily fall in this range. Thus, the sigmoid function is translated and scaled in such a way that,

$$S_u(x, \eta, \sigma, a, b) = \frac{b}{1+\exp(-\frac{x-\eta}{\sigma})} + a, \tag{1}$$
$$S_d(x, \eta, \sigma, a, b) = \frac{b}{1+\exp(\frac{x-\eta}{\sigma})} + a, \tag{2}$$

where a is the lower bound of the function, and b is the range such that the upper bound is given by $a + b$. Figure 1 shows the two switching functions. In our proposed population model, the parameter functions will take the form of one of these modified sigmoid switching functions.

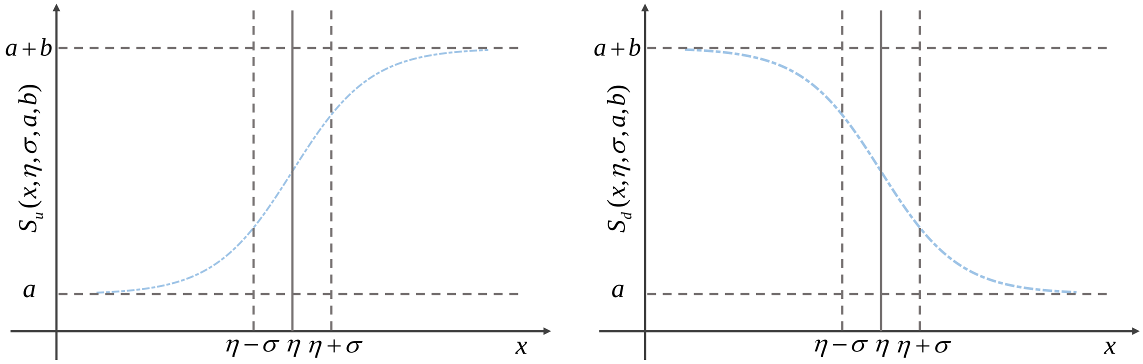

Figure 1: The diagrams of $S_u(x, \eta, \sigma, a, b)$ and $S_d(x, \eta, \sigma, a, b)$

Cost Function Setting

Our cost function is designed to include the hospitalization cost $F_H(t)$, individual opportunity cost due to isolation $F_I(t)$, human capital investment $F_C(t)$ and the cost of risky behavior $F_R(t)$. They are discussed below:

(a) **Hospitalization cost** consists of two parts, severe cases and standard cases cost, i.e.

$$F_H(t) = C_T(1-\chi)D(t) + C_S\chi D(t), \tag{3}$$

where χ is the severe disease rate, taken to be 0.1, according to a report by WHO[2]. C_t and C_s are the hospitalization cost of each standard case and severe case per day, respectively. We take $C_t = 2000$ and $C_s = 20000$, respectively[3].

(b) **Individual opportunity cost due to isolation** takes into account opportunity loss due to the individual under lockdown, i.e.

$$F_I(t) = \begin{cases} I(t) \times \theta \times C_I \times \left(\frac{t-t_{start}}{T_I}\right), \text{ if } t - t_{start} > T_I \\ I(t) \times \theta \times C_I, \text{ if } t - t_{start} < T_I \end{cases} \tag{4}$$

where θ is the proportion of affected people, C_I is the cost per individual in I per day, t_{start} is the time that the lockdown begins, T_I is the duration of the lockdown that causes $F_I(t)$ to change drastically. In this paper, θ is taken to be 0.4, because some industries are still operating despite the COVID-19 situation. For example, medical enterprises, high-technology enterprises, and certain industries are less affected by COVID-19, as are companies that can implement telecommuting arrangement successfully. $C_I = 50$ is taken from[4] and $T_I = 20$.

(c) **Human capital investment** is the cost due to human capital removed from the workforce as a result of individuals in the hospital or have died, that is, D and E. A reduction in human capital has an overall effect on the economic output of the population in S, so

$$F_C(t) = C_D D(t) + C_E E(t), \tag{5}$$

where C_D and C_E are the human capital investment by D and E, respectively. $F_C(t)$ caused by individuals in D and E will affect all population, so it is reasonable for them to be borne by everyone in the population. For illustrative purpose, we take $C_D = 20000$ and $C_E = 25000$. Although these values are large for an individual, it is small when distributed across the entire population.

(d) **Cost of risky behavior** is determined by the number of individuals in A, i.e.

$$F_R(t) = C_R A(t), \tag{6}$$

where C_R is the cost per individual in A per day, and set to be 500 in this paper. The cost here refers to the spread of disease due to an individual who is still in the community despite being a carrier of the disease.

The cumulative cost $\mathcal{F}(t)$ is considered together with the "cost" per day, to show the effectiveness of the different strategies. An effective strategy will avoid a rapid increase in $\mathcal{F}(t)$. Due to the variable step integrator in *ode23* differential equation solver, the cost function $F(t)$ in a day should be divided across an equal number of time intervals first, that is,

$$\bar{F}(i) = \frac{\sum\limits_{t \in X_i} F(t)}{|X_i|}, \tag{7}$$

where i is the ith time interval, and one time interval equals to 10^{-3} in this paper. $|X_i|$ is the number of data points in the ith time interval, and $\bar{F}(i)$ represents the average cost in the ith time interval. Consequently, the cumulative cost $\mathcal{F}(t)$ at time t is defined as follows:

$$\mathcal{F}(t) = \sum_{i=1}^{t} \bar{F}(i). \tag{8}$$

References

[1] M. Kuwamura, T. Nakazawa, T. Ogawa. *Journal of Mathematical Biology* **2009**, *58*, 3 459.

[2] Who report. https://www.who.int/docs/default-source/coronaviruse/situation-reports/20200306-sitrep-46-covid-19.pdf?sfvrsn=96b04adf_4. Last accessed date: 20 June 2020.

[3] National healthcare security administration of the people's republic of china. http://www.gov.cn/xinwen/2020-04/12/content_5501508.htm. Last accessed date: 20 June 2020.

[4] 116th United States Congress. Coronavirus aid, relief, and economic security act, **2020**. URL https://www.congress.gov/bill/116th-congress/house-bill/748/. Last accessed date: 20 June 2020.
